# The FXR Agonist, Obeticholic Acid, Suppresses HCC Proliferation & Metastasis: Role of IL-6/STAT3 Signalling Pathway

**DOI:** 10.1038/s41598-017-12629-4

**Published:** 2017-10-02

**Authors:** Yasmeen M. Attia, Rasha A. Tawfiq, Aya A. Ali, Mohamed M. Elmazar

**Affiliations:** Department of Pharmacology, The Center for Drug Research and Development (CDRD), Faculty of Pharmacy, The British University in Egypt, El Sherouk City, P.O. Box 43, Cairo, 11837 Egypt

## Abstract

The nuclear receptor, farnesoid X receptor (FXR), has been recently considered as a tumor suppressor in HCC. IL-6/Janus kinase 2 (Jak-2)/signal transducer and activator of transcription 3 (STAT3) pathway has been implicated as a key player in many cancer types. This study aimed at investigating the potential effect of the FXR agonist, obeticholic acid (OCA), on HCC and the involvement of IL-6/STAT3 pathway. The potential regulation of STAT3 by its main feedback inhibitor target gene, the suppressor of cytokine signaling 3 (SOCS3), triggered by OCA was also explored. Cytotoxicity studies were performed on HepG2, Huh7, and SNU-449 cell lines using OCA alone and combined with the FXR antagonist guggulsterone (Gugg). OCA cytotoxic effect was significantly hampered in presence of Gugg. OCA also caused cell cycle arrest and inhibited invasion and migration of HCC cells. Decrease in STAT3 phosphorylation and SOCS3 upregulation were also observed. Moreover, Jak-2, IL-1β, and IL-6 levels were decreased. These results were correlated with an upregulation of FXR and small heterodimer partner (SHP) levels. Effects of OCA on IL-6/STAT3 main key players were reversed in presence of Gugg. Overall, these findings suggest a potential effect of OCA in HCC *via* interfering with IL-6/STAT3 signalling pathway *in vitro*.

## Introduction

Hepatocellular carcinoma (HCC) is the sixth most prevalent type of cancer and the third most common cause of cancer-related death worldwide. It accounts for about 85% of primary liver cancer where its incidence has dramatically increased during the past two decades^1^. Previous studies showed that the molecular pathogenesis of HCC comprises a multi-step process that subsequently leads to the accumulation of various aberrant genetic and epigenetic changes^2^.

The Farnesoid X Receptor (FXR) is a nuclear receptor that has long been considered as a main regulator of metabolism with multifaceted functions in the maintenance of bile acids, lipids, and glucose homeostasis^3–5^. Recently, FXR agonists were also found to exert tumor suppression properties in liver tissue through various mechanisms^6–8^.

It was clearly stated in previous studies that deficiency of FXR contributes to hepatic carcinogenesis, though the underlying mechanisms haven’t yet been totally investigated and could be either FXR-dependent or FXR-independent. FXR deficiency is known to lead to cholestasis^9,10^, which subsequently causes hepatic injury and inflammation^11,12^. It is suggested that inflammation is a pivotal mechanism contributing to hepatic carcinogenesis^13^. One of the main mechanisms which can explain how inflammation leads to HCC is the activation of IL-6/Janus kinase 2 (Jak-2)/signal transducer and activator of transcription 3 (STAT3) signalling pathway, which is considered a potent promoting factor driving tumor formation in the liver^14^. STAT3 activation turns on the expression of a cohort of genes that further induce cell division and inhibit apoptosis^14^. Moreover, STAT3 remarkably induces the gene expression of suppressor of cytokine signalling 3 (SOCS3), which leads to a feedback suppression of STAT3 activity^15,16^. There is a growing body of evidence suggesting that FXR deficiency causes STAT3 activation; however, the underlying mechanism is not yet clear.

Obeticholic acid (OCA), also known as INT-747 or 6α-ethyl-chenodeoxycholic acid, is a first-in-class bile acid analogue, derived from chenodeoxycholic acid, the natural FXR agonist, and shows almost 100-fold greater FXR agonistic activity than chenodeoxycholic acid^17^ without activating other nuclear receptors^18^. Experimentally, OCA was found to treat hepatic cholestasis and ameliorate hepatic steatosis and insulin resistance^19–21^. OCA is currently in phase I clinical trials for the treatment of alcoholic hepatitis^22^, phase II clinical trials for non-alcoholic steatohepatitis (NASH)^23^ and type-2 diabetes mellitus^21^ as well as phase III clinical trials for primary biliary cirrhosis (PBC)^22^. It’s worth mentioning that OCA has also been recently approved by the FDA as an orphan drug for the use in PBC^24^.

The present study aimed at investigating the effect of OCA on the proliferation and metastasis of HCC cells providing further insights into the possible underlying mechanisms, particularly the involvement of IL-6/STAT3 signalling axis.

## Material and Methods

### Cell lines and cell culture

HCC cell lines; HepG2, Huh7, and SNU-449 as well as THLE-2 normal adult liver cells were obtained from the American Type Culture Collection (ATCC, VA, USA). Cells were cultured using Dulbecco’s Modified Eagle Medium (DMEM; Invitrogen, Life Technologies, CA, USA) supplemented with 10% fetal bovine serum (FBS; GE Healthcare HyClone, WV, USA), streptomycin (100 μg/ml), and penicillin (100 units/ml) in a humidified atmosphere of 5% CO_2_ at 37 °C. Cells were serially passaged at 80–90% confluency.

### Cytotoxicity assay

HepG2, Huh7, and SNU-449 cells were seeded in 96-well plates. Twenty-four hours later, cells were treated with OCA or vehicle; Dimethylsulphoxide (DMSO; Sigma-Aldrich, St. Louis, MO, USA). Cytotoxicity test was performed using 3-(4,5-dimethylthiazol-2-yl)-2,5-diphenyltetrazolium bromide (MTT) assay (Sigma-Aldrich, St. Louis, MO, USA) according to the manufacturer’s instructions, as previously described^25^. Briefly, 1.2 to 1.8 × 10^3^ cells per well were seeded in 96-well plates and cultured overnight. Then, the cells were treated with 0.01-100 μM of OCA (eNovation Chemicals LLC, USA), OCA (0.01–100 µM) +10 µM guggulesterone (Gugg; FXR antagonist; Sigma Aldrich, USA), or DMSO. After 72 h, MTT reagent was added to the cells in each well followed by incubation for 2 h, and the absorbance was determined using a microplate reader. The effect of treatments on cell viability was presented as the percentage of the absorbance at 570 nm from treated cells versus that from untreated control cells. All experiments were performed in triplicates. Half-maximal inhibitory concentration (IC_50_) was calculated using GraphPad Prism software, version 5.00 (GraphPad Software, CA, USA) according to the non-linear regression fitting model.

### Apoptosis assay using Annexin V/Propidium iodide staining

HepG2 and Huh7 cells cultured in six-well plates were treated with OCA for 48 hours. Then, the cells were fixed in cold ethanol for half an hour and stained with 5 μl Annexin V-FITC and 5 μl propidium iodide (PI) using an Annexin V‑FITC Apoptosis Detection Kit (BioVision, CA, USA). The cells were then placed at room temperature for 15 min in the dark then analyzed by a FACScan Flow Cytometer (Beckman Coulter, CA, USA). Apoptosis was evaluated in terms of the FITC-positive cells.

### Cell cycle analysis

After 48 h exposure of HepG2 & Huh7 cells to OCA, cells were trypsinized and washed with PBS, resuspended in cold methanol, and kept overnight at 4 °C. Collected cells were then resuspended in 250 μl of 1.12% sodium citrate buffer (pH 8.4) together with 12.5 μg RNase, and incubated at 37 °C for 30 min. After centrifugation, cells were resuspended in PBS and filtered. Cell cycle analysis was performed using FACSCalibur Flow Cytometer (BD Biosciences, San Jose, CA, USA).

### Migration and invasion assays

To assess cell migration, HepG2 and Huh7 cells (5 × 10^5^ cells/well) were seeded on top of the 8.0 μm pore size transwell insert containing 600 μl DMEM medium in the lower chamber. Cells migrated to the other side of membrane were stained with crystal violet and counted after 24 h incubation. To assess cell invasion, cells (1 × 10^5^) were plated in 8.0 μm pore size Matrigel coated transwell insert. Culture medium was changed twice a day for 3 days. After 48 h, cells that had migrated through the Matrigel to the filter were stained with crystal violet and counted.

### Experimental design

HepG2 or Huh7 cells were divided into three groups: (1) Blank control group where cells were treated with the vehicle; DMSO, (2) OCA group where cells were treated with IC_50_ of OCA; & (3) OCA+ Gugg group where cells were treated with both IC_50_ of OCA & 10 µM of Gugg to achieve FXR receptor blockade. All treatments were started 24 hrs after cells were seeded in T-25 flasks. Gene expression levels were assessed at 48 h of treatment exposure, while protein expression levels were assessed at 72 h of exposure. Moreover, the expression of a number of genes (IL-1β, IL-6, STAT3, & FXR) was estimated in THLE-2 normal liver cells for comparison with their corresponding expression in HCC cells.

### RNA extraction and real time quantitative polymerase chain reaction (RT-qPCR) assay

Total RNA was isolated using RNeasy Mini Kit (Qiagen, USA). Complementary DNA (cDNA) synthesis and PCR amplification were carried out using iScript^TM^ One-Step RT-PCR Kit with SYBR^®^ Green (Bio-Rad, CA, USA) according to the manufacturer’s instructions using GAPDH as the house-keeping gene, as described previously^26^. The relative mRNA expression levels (fold change from untreated control samples normalized to GAPDH as the housekeeping gene) of caspase-3, FXR, small heterodimer partner (SHP), STAT3, SOCS3, Jak-2, IL-1β, and IL-6 were assessed using the 2^−ΔΔCt^ analysis method, as previously described by Livak and Schmittgen^27^ for all experiments except those performed on THLE-2 cells for estimating the expression of genes in normal cells *vs* HCC cells. For the latter experiment, absolute quantitation method was used as described by Fronhoffs *et al*.^28^. The primer sequences used for RT-qPCR are listed in Table 1.

**Table 1:** Sequence of primers used for quantitative real time polymerase chain reaction (qPCR) and their National Center for Biotechnology Information (NCBI) accession numbers.

Primer	Sequence	NCBI Accession number
Caspase-3	Forward: 5′-TTCATTATTCAGGCCTGCCGAGG-3′	NM_032991.2
	Reverse: 5′-TTCTGACAGGCCATGTCATCCTCA-3′	
FXR	Forward: 5′-ACCAGCCTGAAAATCCTCAACAC-3′	NM_001206993.1
	Reverse: 5′-CTCTCCATGACATCAGCATCTCAG-3′	
SHP	Forward: 5′-AGGGACCATCCTCTTCAACC-3′	NM_021969.2
	Reverse: 5′-TTCACACAGCACCCAGTGAG-3′	
STAT3	Forward: 5′-AGCATCCTGAAGCTGACCCAGGT-3′	NM_139276.2
	Reverse: 5′-TCGGCAGGTCAATGGTATTGCTGC-3′	
SOCS3	Forward: 5′-ATCCTGGTGACATGCTCCTC-3′	NM_003955.4
	Reverse: 5′-GGCACCAGGTAGACTTTGGA-3′	
Jak-2	Forward: 5′-GGGAGGTGGTCGCTGTAAAA-3′	NM_001322204.1
	Reverse: 5′-ACCAGCACTGTAGCACACTC-3′	
IL-1β	Forward: 5′-AATCTGTACCTGTCCTGCGTGTT-3′	NM_000576.2
	Reverse: 5′-TGGGTAATTTTTGGGATCTACACTCT-3′	
IL-6	Forward: 5′-TGCCAGCCTGCTGACGAAG-3′	NM_001318095.1
	Reverse: 5′-AACAATCTGAGGTGCCCATGCTAC-3′	
GAPDH	Forward: 5′-GGATTTGGTCGTATTGGG-3′	NM_001289746.1
	Reverse: 5′-GGAAGATGGTGATGGGATT-3′	

### Determination of caspase-3 activity

Active caspase-3 level was measured using human active Caspase-3 ELISA kit (Invitrogen, CA, USA) according to the manufacturer’s instructions, in order to investigate the effect of OCA on apoptosis. Briefly, cells were washed with PBS, collected and added to extraction buffer containing protease inhibitor (1 ml per 1 × 10^7^ cells) then diluted immediately prior to the assay. After completing the steps of the assay, the optical density was measured for each well at 450 nm.

### Determination of SOCS3, total STAT3, and phosphorylated STAT3 activity

To determine the effect of OCA on SOCS3, total STAT3 (t-STAT3), and phosphorylated STAT3 (p-STAT3) activity; the human SOCS3 ELISA kit (Cloud-Clone Corp., TX, USA) and the ELISA kit for the determination of t-STAT3 and p-STAT3 (Tyr705) (Abcam, MA, USA) were used according to the manufacturers’ instructions. Briefly, cells were washed with PBS, collected and added to the extraction buffer containing protease inhibitors (1 ml per 1 × 10^7^ cells) then diluted immediately prior to the assay. After performing all steps of the assay, the optical density of each well was determined within 30 minutes using a microplate reader set at 450 nm.

### Statistical analysis

All values are presented as means ± standard deviation (S.D.) from three independent experiments performed in triplicates. 95% confidence intervals (C.I.) were compared to test significance for the fold changes from control untreated samples in gene expression studies. Statistical analysis was performed by Student’s t-test and one-way ANOVA using GraphPad Prism, version 5.00 (GraphPad Software, CA, USA) (v5). Statistical significance was determined at P < 0.05.

### Data availability

The datasets generated during and/or analysed during the current study are available from the corresponding author on reasonable request.

## Results

### Obeticholic acid inhibits HCC cell growth and induces cell cycle arrest

**Figure 1:**
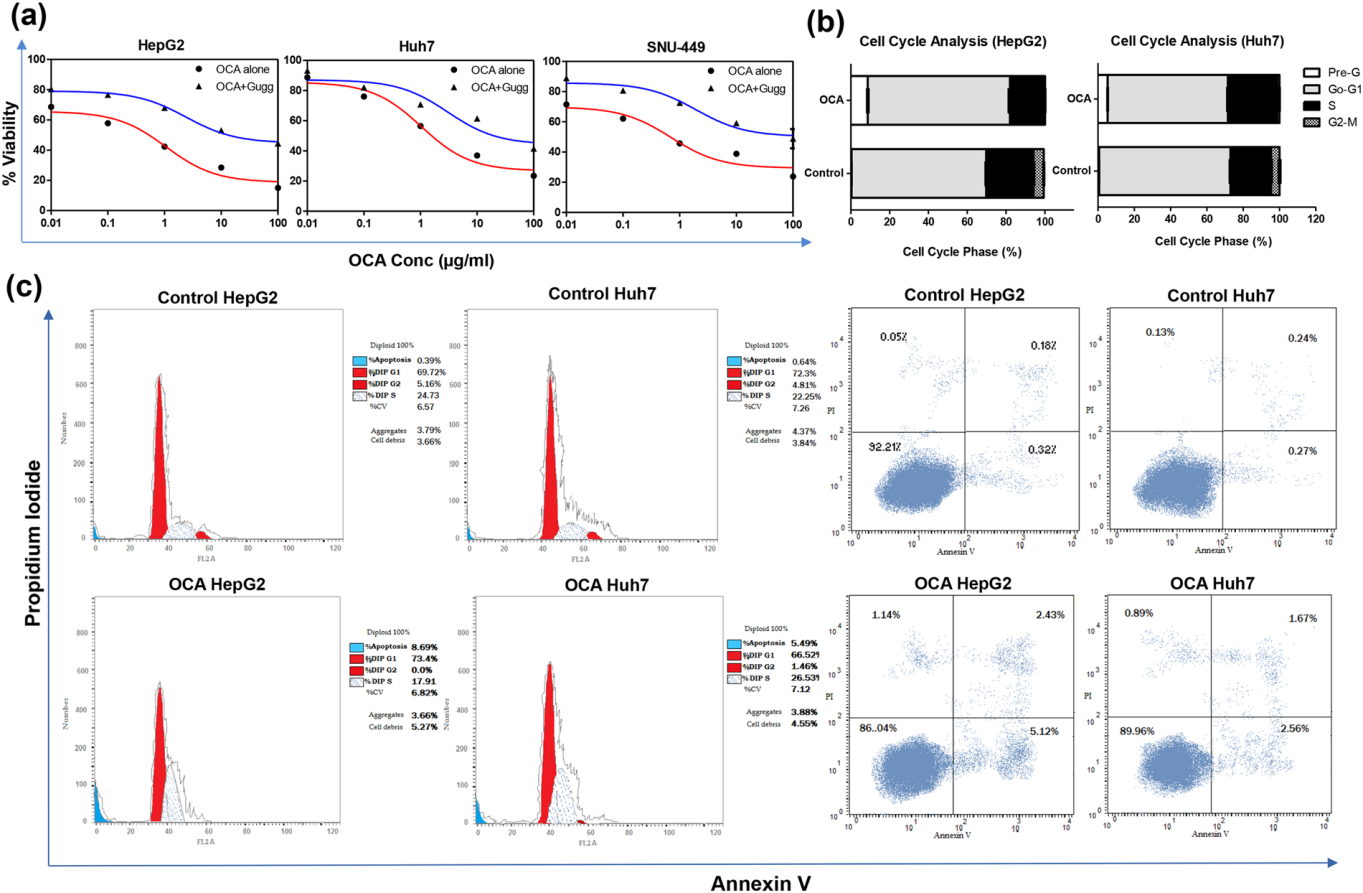
Effect of OCA on viability and cell cycle progression in HCC cell lines. (**a**) Dose-response plots of OCA, alone and combined with the FXR antagonist, Gugg, on HepG2, Huh7, and SNU-449 cell lines after 72 h exposure, as detected by MTT assay. **(b**,**c)** DNA content-based cell cycle analysis in HepG2 and Huh7 cell lines treated with OCA. Results represent three independent experiments performed in triplicates.

MTT assay showed that OCA decreased the rate of cell proliferation in HepG2, Huh7, and SNU-449 cell lines, as compared to the corresponding control (Fig. 1). The IC_50_ of OCA was determined and found to be 1.067 μM, 1.038 μM, and 0.706 µM on HepG2, Huh7, and SNU-449 cell lines, respectively. However, Gugg caused a 2.24-, 2.95-, and 2.76-fold increase in the IC_50_ of OCA in HepG2, Huh7, and SNU-449 cell lines, respectively. Moreover, as shown in Fig. 1, cell cycle analysis showed that OCA caused a 1.06-fold increase in the number of HepG2 cells at G0/G1 phase with a significant decrease in the cells in S phase by 26.13% (P < 0.0001), as compared to control. Whereas in Huh7 cell line, OCA caused cell growth arrest at the S phase where accumulated cells reached 21.18% (P = 0.0003), as compared to untreated cells. Moreover, the cells in M phase were 3.51-fold lower (P = 0.0005) than control in Huh7 cell line. Interestingly, OCA has also caused a 23.68- and 8.32-fold increase (P < 0.0001) in the percentage of cells in the pre-apoptotic phase in HepG2 and Huh-7 cell lines, respectively.

### Obeticholic acid inhibits cell migration and invasion of HCC cells

**Figure 2:**
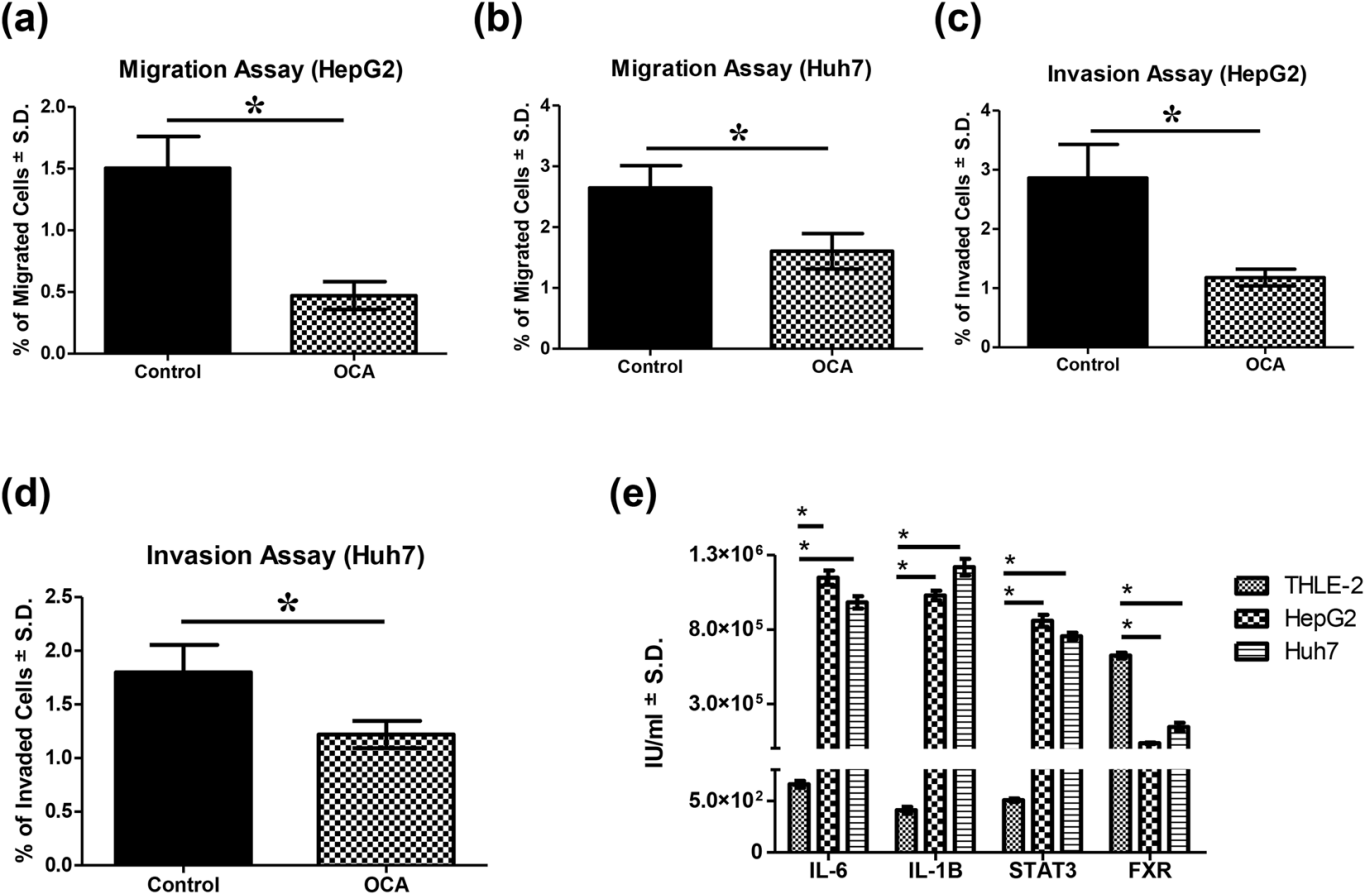
Migration and invasion assays for OCA on HCC cell lines & gene expression in normal cells *vs* HCC cells. Migration assays performed on **(a)** HepG2 and **(b)** Huh7 cell lines. Invasion assays performed on **(c)** HepG2 and **(d)** Huh7 cell lines. **(e)** Gene expression of IL-6, IL-1β, STAT3, & FXR in THLE-2 normal liver cells, HepG2, & Huh7 cells. Values are presented as means ± S.D. from three independent experiments performed in triplicates. Gene expression levels were estimated using qPCR absolute quantitation method. Statistical analysis was performed using Student’s t test for migration and invasion assays and one-way ANOVA for gene expression analysis. *Significantly different (at P < 0.05) versus control untreated cells (**a**–**d**) or normal liver cells (**e**).

Transwell migration and Matrigel assays were used to investigate the effect of OCA on both migration and invasion, respectively, in HepG2 and Huh7 cell lines. As shown in Fig. 2, OCA caused 3.19- (P = 0.0031) and 1.65- (P = 0.0181) fold decrease in the number of migrated cells in HepG2 and Huh7 cell lines treated with OCA, as compared to controls, respectively. Moreover, OCA caused 2.43- (P = 0.0075) and 1.48- (P = 0.0245) fold decrease in the invasion percentage in HepG2 and Huh7 cell lines, respectively.

### Upregulation of IL-1β, IL-6, & STAT3 and downregulation of FXR in HCC cells *vs* normal cells

In order to validate an altered IL-6/STAT3 axis as well as FXR deficiency in HCC cells, the gene expression of IL-1β, IL-6, STAT3, & FXR was assessed in normal liver cells and compared to expression levels in HepG2 and Huh7 cells. As shown in Fig. 2, the expression levels of IL-1β, IL-6, and STAT3 were highly significantly increased (P < 0.0001) in both HepG2 and Huh7 cells, as compared to normal liver cells. Moreover, FXR expression was highly significantly downregulated (P < 0.0001) in HepG2 and Huh7 cells versus normal cells.

### Obeticholic acid increases caspase-3 gene expression and protein levels

**Figure 3:**
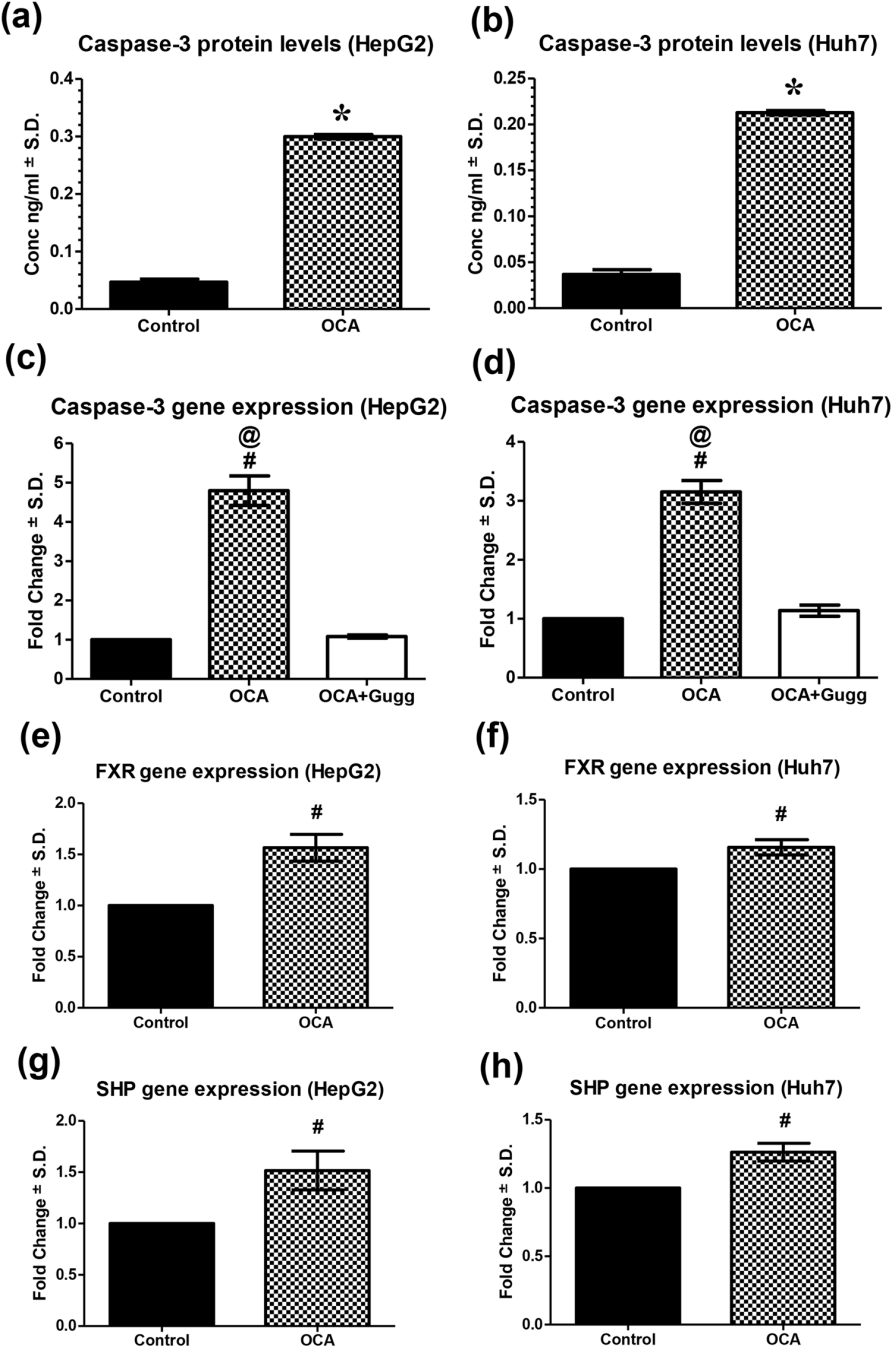
Effect of OCA on caspase-3, FXR, and SHP expression levels. Caspase-3 protein expression on **(a)** HepG2 and **(b)** Huh7 cell lines treated with OCA. Caspase-3 gene expression levels in OCA- and OCA+Gugg-treated **(c)** HepG2 and **(d)** Huh7 cell lines. FXR gene expression on **(e)** HepG2 and **(f)** Huh7 cell lines treated with OCA. SHP gene expression on **(g)** HepG2 and **(h)** Huh7 cell lines treated with OCA. Protein levels were estimated using ELISA. Gene expression levels were estimated using relative qRT-PCR method (fold change from control untreated samples normalized to GAPDH). Values are presented as means ± S.D. from three independent experiments performed in triplicates. *P < 0.05 significant from control untreated cells using Student’s t test. ^#^P < 0.05 significant when the 95% C.I. was compared with control untreated cells. ^@^P < 0.05 significant when the 95% C.I. was compared with OCA+Gugg-treated cells.

To assess the effect of OCA on apoptosis, caspase-3 levels were estimated on both gene and protein levels. OCA caused 6.39- and 5.78-fold increase (P < 0.0001) in active caspase-3 protein levels, as compared to control, in HepG2 and Huh7 cell lines, respectively (Fig. 3a,b). Moreover, it was found that OCA induced upregulation of caspase-3, as determined by the relative mRNA levels, causing 4.80- (95% C.I.: 3.87 to 5.73) and 3.15- (95% C.I.: 2.67 to 3.64) fold increase, in both HepG2 and Huh7 cells, respectively (Fig. 3c,d). These effects were opposed in presence of the FXR antagonist, Gugg, where the fold change expression levels weren’t significantly different from control (95% C.I.: 0.91 to 1.38).

### Obeticholic acid upregulates FXR and its main target gene SHP in HCC cells

To investigate the ability of OCA to activate FXR in HCC cells and test whether this activation is related to the antitumour effects observed, mRNA levels of FXR and its main target gene, SHP, were measured using qRT-PCR. As shown in Fig. 3e and 3f, FXR was upregulated in both HepG2 and Huh7 cells treated with OCA reaching 1.57- (95% C.I.: 1.24 to 1.89) and 1.16- (95% C.I.: 1.02 to 1.30) fold increase from control, respectively. Additionally, OCA caused 1.52- (95% C.I.: 1.05 to 1.99) and 1.27- (95% C.I.: 1.11 to 1.43) fold higher relative mRNA SHP expression levels in HepG2 and Huh7 cell lines, respectively.

### FXR activation by obeticholic acid represses STAT3 activation in HCC cells

**Figure 4:**
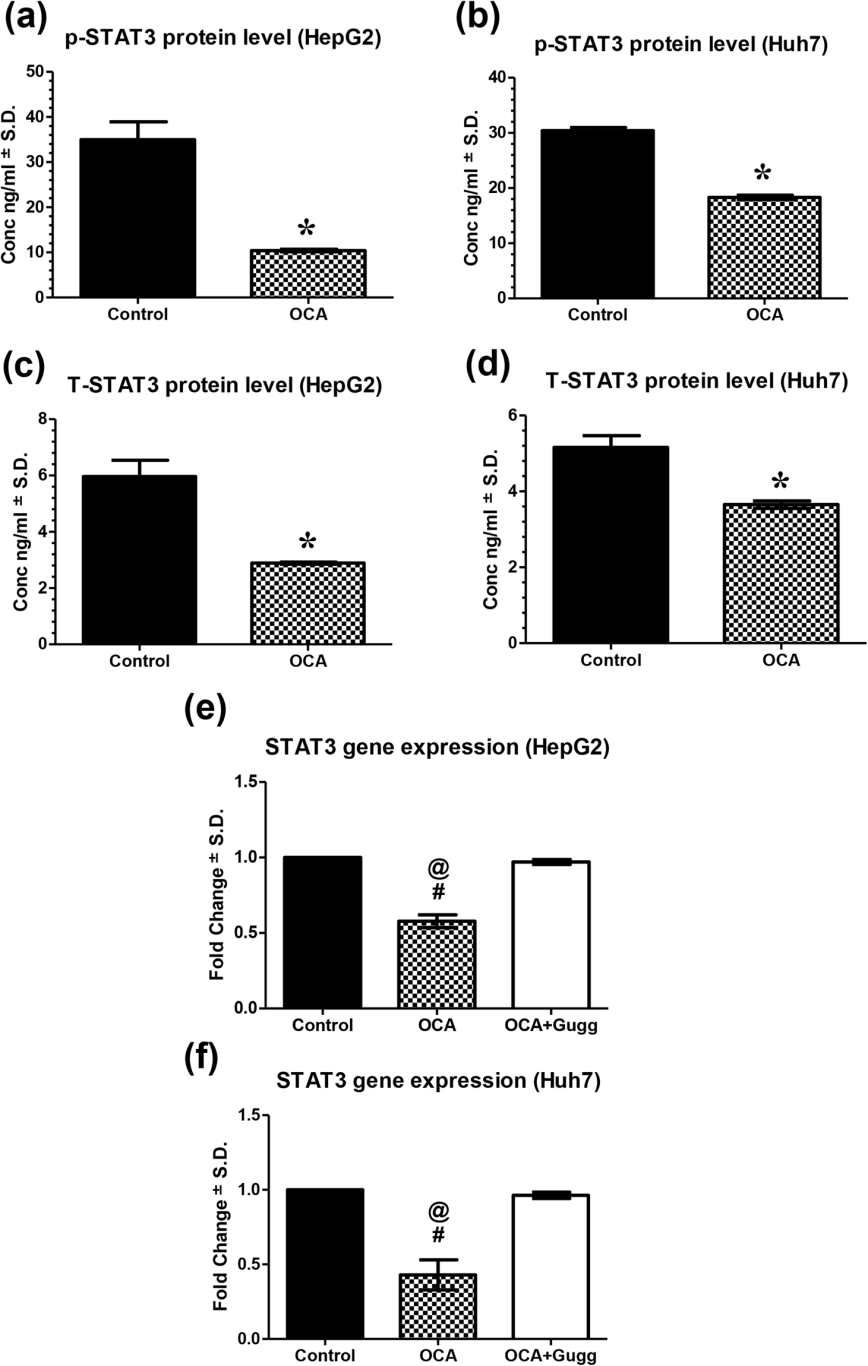
Effect of OCA on STAT3 expression levels. Phosphorylated STAT3 (p-STAT3) protein expression levels in OCA-treated **(a)** HepG2 and **(b)** Huh7 cell lines. Total STAT3 (t-STAT3) protein expression levels in OCA-treated **(c)** HepG2 and **(d)** Huh7 cell lines. STAT3 gene expression on **(e)** HepG2 and **(f)** Huh7 cell lines treated with OCA alone or OCA+Gugg. Protein levels were estimated using ELISA. Gene expression levels were estimated using relative qRT-PCR method (fold change from control untreated samples normalized to GAPDH). Values are presented as means ± S.D. from three independent experiments performed in triplicates. *P < 0.05 significant from control untreated cells using Student’s t test. ^#^P < 0.05 significant when the 95% C.I. was compared with control untreated cells. ^@^P < 0.05 significant when the 95% C.I. was compared with OCA+Gugg-treated cells.

As a result of FXR activation by OCA, STAT3 activation was consequently reduced, as assessed by the amount of p-STAT3 (Tyr705) and t-STAT3 protein. As shown in Fig. 4, both p-STAT3 and t-STAT3 were reduced in OCA-treated HepG2 cells, compared to untreated cells, by 70.24% (P = 0.0004) and 51.56% (P = 0.0008), respectively. Moreover, p-STAT3 and t-STAT3 were also reduced in OCA-treated Huh7 cells by 39.70% (P < 0.0001) and 29.19% (P = 0.0013), respectively. Similarly, STAT3 relative mRNA levels, were 1.73- (95% C.I.: 0.47 to 0.68) and 2.33- (95% C.I.: 0.18 to 0.68) fold lower in HepG2 and Huh7-OCA-treated cells than control untreated cells, respectively. This latter effect was opposed in cells treated with OCA and Gugg where the expression wasn’t significantly changed from control, either in HepG2 (95% C.I.: 0.93 to 1.01) or Huh7 cells (95% C.I.: 0.91 to 1.02).

### FXR activation by obeticholic acid induces SOCS3 expression levels

**Figure 5:**
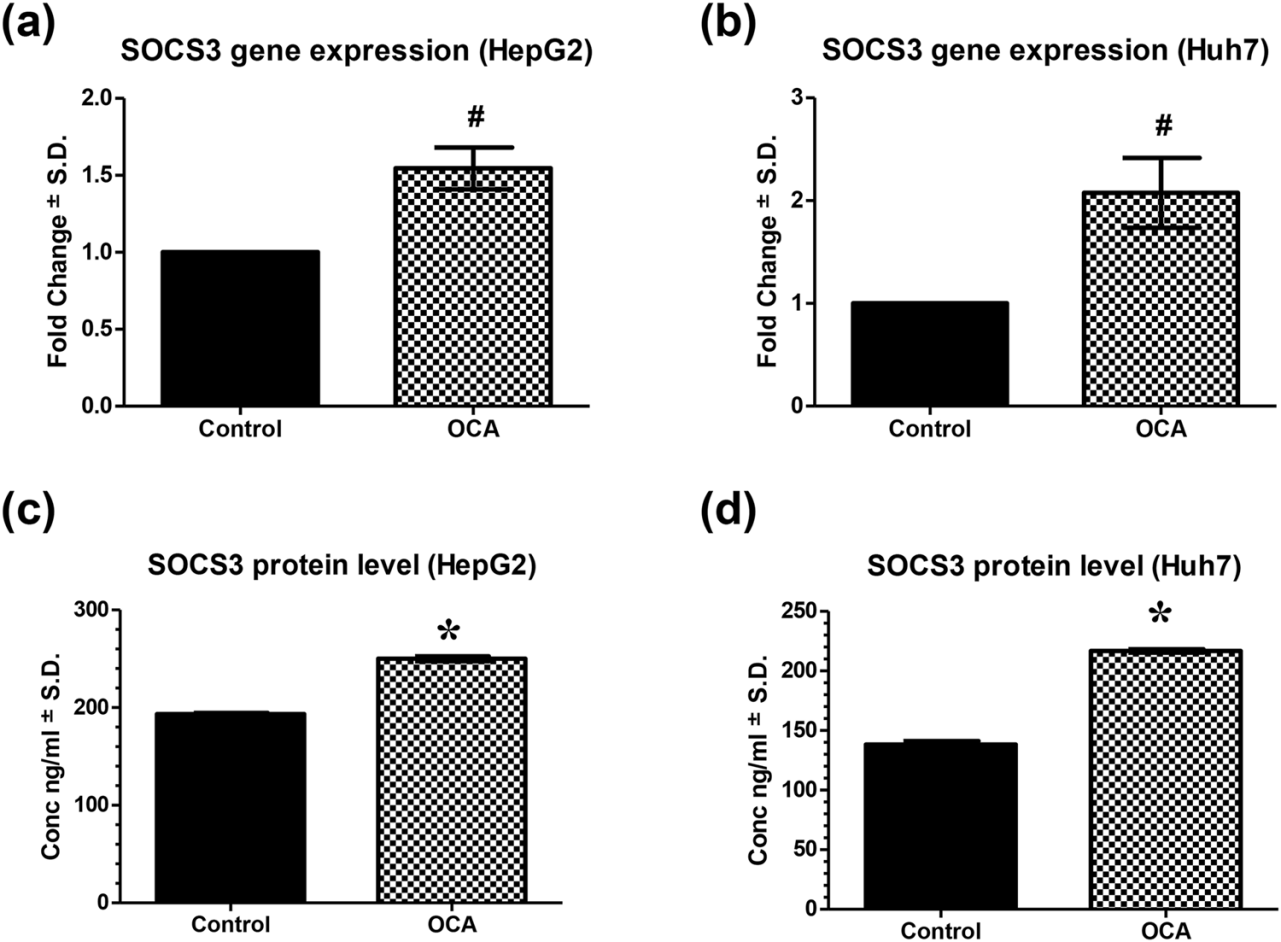
Effect of OCA on SOCS3 expression levels. SOCS3 gene expression on **(a)** HepG2 and **(b)** Huh7 cell lines treated with OCA. SOCS3 protein expression levels in OCA-treated **(c)** HepG2 and **(d)** Huh7 cell lines. Protein levels were estimated using ELISA. Gene expression levels were estimated using relative qRT-PCR method (fold change from control untreated samples normalized to GAPDH). Values are presented as means ± S.D. from three independent experiments performed in triplicates. *P < 0.05 significant from control untreated cells using Student’s t test. ^#^P < 0.05 significant when the 95% C.I. was compared with control untreated cells.

In order to determine whether FXR activation by OCA resulted in subsequent changes in SOCS3, one of the FXR target genes and also an important feedback repressor of STAT3, we examined the gene and protein expression profiles of SOCS3 in OCA-treated HepG2 and Huh7 cells. As shown in Fig. 5, relative mRNA levels of SOCS3 were 1.55- (95% C.I.: 1.21 to 1.88) and 2.08- (95% C.I.: 1.23 to 2.92) fold higher in HepG2 and Huh7 OCA-treated cells compared to controls, respectively. This was also reflected on the protein level, where SOCS3 showed 1.29- and 1.56-fold higher expression levels in HepG2 and Huh7 cells (P < 0.0001), respectively.

### Obeticholic acid inhibits the activation of Jak-2

**Figure 6:**
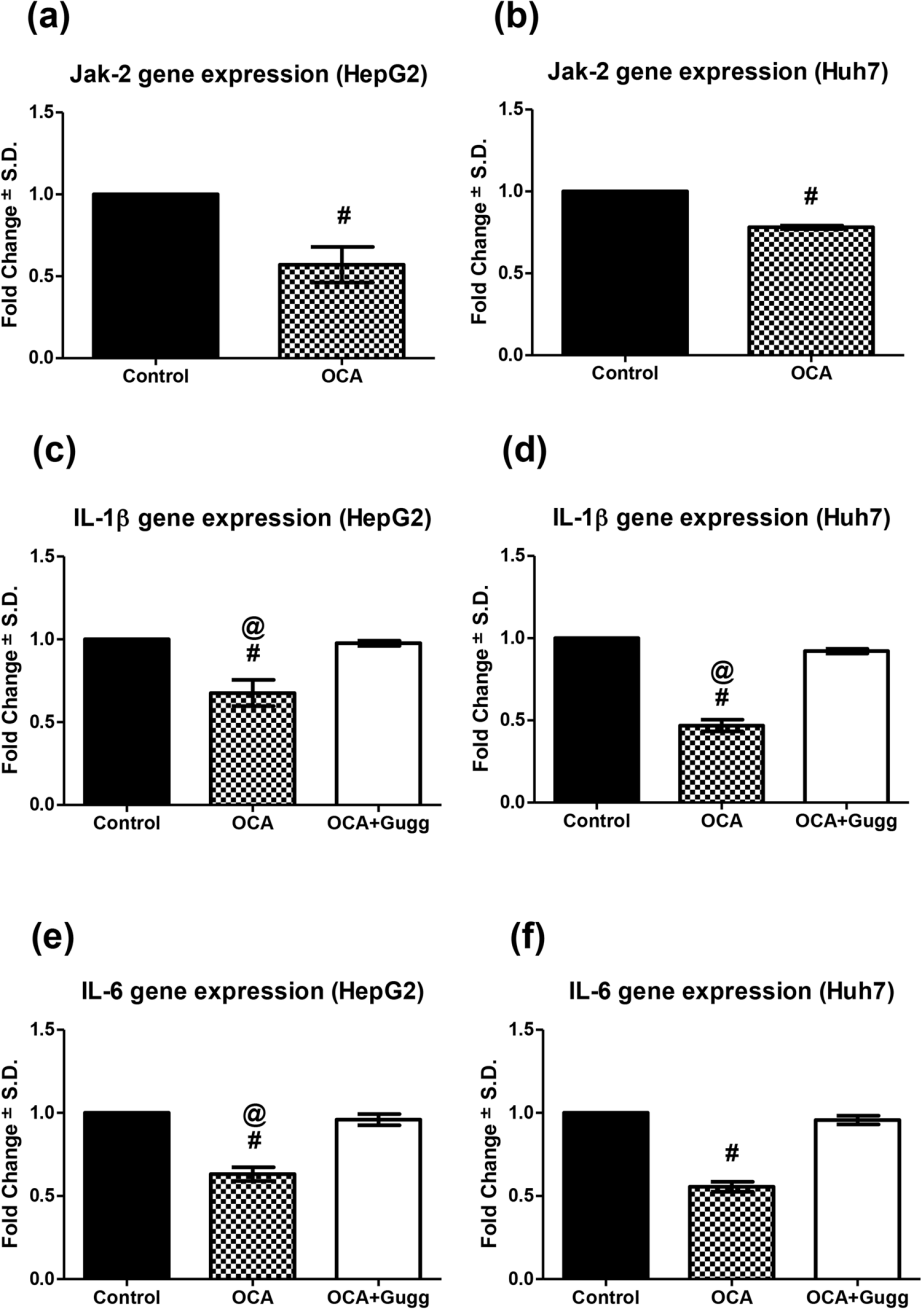
Effect of OCA on Jak-2, IL-1β, and IL-6 expression levels. Jak-2 gene expression on **(a)** HepG2 and **(b)** Huh7 cell lines treated with OCA. IL-1β gene expression on **(c)** HepG2 and **(d)** Huh7 cell lines treated with OCA or OCA+Gugg. IL-6 gene expression on **(e)** HepG2 and **(f)** Huh7 cell lines treated with OCA or OCA+Gugg. Gene expression levels were estimated using relative qRT-PCR method (fold change from control untreated samples normalized to GAPDH). Values are presented as means ± S.D. from three independent experiments performed in triplicates. ^#^P < 0.05 significant when the 95% C.I. was compared with control untreated cells. ^@^P < 0.05 significant when the 95% C.I. was compared with OCA+Gugg-treated cells.

To further confirm the inhibition of the IL-6/STAT3 signalling pathway by OCA and whether the increase in SOCS3 had subsequently caused an inhibition of Jak-2, the relative mRNA level of Jak-2 in OCA-treated cells was determined. As shown in Fig. 6a and 6b, Jak-2 mRNA level was 1.75- (95% C.I.: 0.30 to 0.84) and 1.28- (95% C.I.: 0.76 to 0.81) fold lower in HepG2 and Huh7 cells treated with OCA, respectively, as compared to control untreated cells.

### FXR activation by obeticholic acid inhibits IL-1β and IL-6 expression levels

Since FXR deficiency reported in HCC was associated with increased levels of IL-1β and IL-6, we were interested in investigating the potential influence of OCA on their expression levels in the present experiment. Interestingly, we found that both IL-1β and IL-6 gene expression levels were 1.48- (95% C.I.: 0.48 to 0.87) and 1.58- (95% C.I.: 0.53 to 0.73) fold lower in OCA-treated HepG2 cells as well as 2.13- (95% C.I.: 0.38 to 0.56) and 1.80- (95% C.I.: 0.48 to 0.63) fold lower in OCA-treated Huh7 cells, respectively, compared to untreated control cells. The aforementioned effects were reversed in OCA+Gugg-treated HepG2 cells (95% C.I.; IL-1β: 0.94 to 1.02 & IL-6: 0.88 to 1.04) as well as Huh7 cells (95% C.I.; IL-1β: 0.89 to 0.96 & IL-6: 0.89 to 1.02) (Fig. 6c–f).

## Discussion

**Figure 7:**
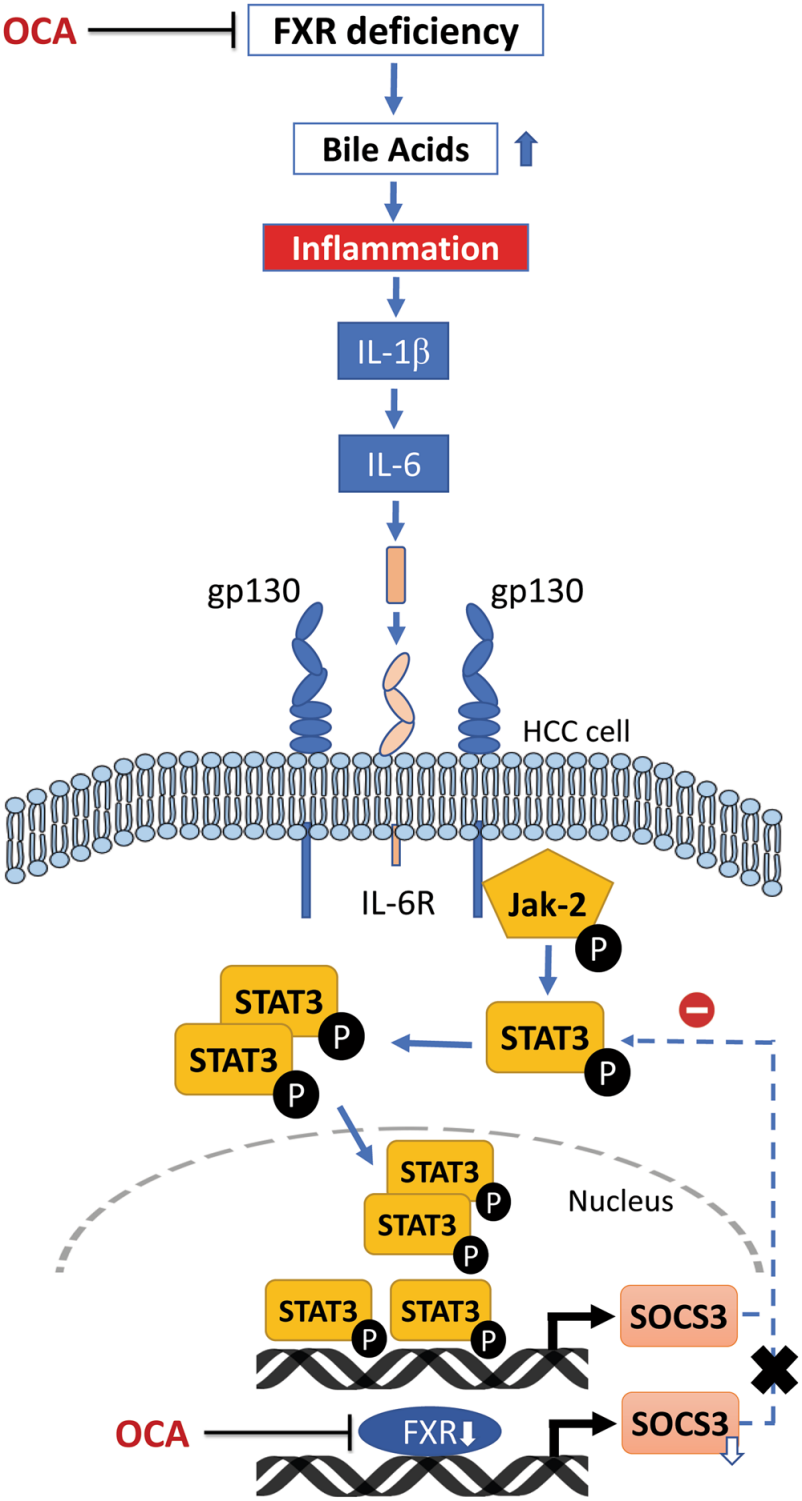
Schematic diagram of the proposed mechanism by which OCA may modulate the IL-6/Jak-2/STAT3 signalling pathway in HCC cells. FXR deficiency leads to activation of STAT3 pathway by increasing IL-1β which subsequently increases IL-6 levels. IL-6 then binds to IL-6 receptor (IL-6R) leading to Jak-2 phosphorylation *via* glycoprotein 130 (gp130) consequently activating STAT3 phosphorylation. STAT3 homodimers are then formed and translocated to the nucleus leading to the activation of many downstream target genes responsible for hepatic carcinogenesis such as SOCS3, the FXR target gene and the feedback inhibitor of STAT3.

The nuclear receptor FXR has long been known for its ability to maintain both glucose and lipid homeostasis in the liver. Recently, the role of FXR in the liver has gone beyond its function as a metabolic regulator and was extended to include liver carcinogenesis. Hence, it is presented as a promising new therapeutic target in HCC where a tumor suppressor potential of FXR agonists in the liver has been reported both *in vivo* and *in vitro* ^29^. Multiple mechanisms have been suggested to explain this antitumour effect^30–32^. One important mechanism is interfering with the activation of IL-6/Jak-2/STAT3 signalling pathway which was found to have a pivotal role in cancer development and progression^30^. OCA, a potent FXR agonist which has recently been approved by the FDA as an orphan drug for PBC is still undergoing clinical trials for indications closely related to the ability of FXR to maintain bile acid, lipid, and glucose levels in the liver such as NASH^23^. The present study provides the first evidence for a potential anticancer effect of OCA in HCC providing insights into the possible underlying mechanism of action. As shown from the results, activation of FXR by OCA was found to suppress HCC cells proliferation, migration, and invasion. The cytotoxic effect of OCA on HepG2, Huh7, and SNU-449 cell lines was compromised in presence of the FXR antagonist, Gugg. In order to assess the effect of OCA on inducing apoptosis of HCC cells, caspase-3 gene and protein expression levels were determined. It was found that OCA significantly reduced caspase-3 in both HepG2 and Huh7 cell lines. In the present study, OCA was also found to inhibit the activation of IL-6/STAT3 signalling pathway in HCC cell lines (Fig. 7). Our results also showed that OCA was capable of causing a significant repression of important key players in this signalling pathway such as IL-6, IL-1β, and Jak-2 besides STAT3 phosphorylation. As previously reported by Li *et al*.^30^, FXR deficiency observed in HCC^6^, was found to activate STAT3 pathway by increasing IL-1β expression which subsequently increased IL-6 levels in liver tissue. IL-6 increased levels led to Jak-2 phosphorylation which consequently activated STAT3 Tyr705 and Ser727 phosphorylation. Following this activation step, STAT3 homodimers are formed and translocated to the nucleus leading to the activation of many downstream target genes related to liver cancer development. Among these genes is the SOCS3 which is considered a very important direct FXR downstream target gene in addition to its pivotal role as a feedback inhibitor of the STAT3 pathway^15,16^. Interestingly, in the current study OCA was found to significantly increase the expression of SOCS3 on both gene and protein levels which, therefore, could have suppressed STAT3 phosphorylation. Upregulation of SOCS3 in response to FXR agonists has been previously reported by Xu *et al*.^33^. In the present experiment, activation of FXR in HCC cell lines by OCA was verified by evaluating the gene expression profile of FXR and its target gene, SHP. As further explained below, OCA caused an upregulation in both FXR and SHP mRNA levels that hence could be correlated to changes observed in STAT3 mRNA and protein levels. FXR was previously reported to directly induce the expression of the HCC suppressor, SHP^34^.

Interestingly, the effects of OCA on IL-1β, IL-6, STAT3, and caspase-3 have been reversed in presence of the FXR antagonist, Gugg, suggesting a causative relationship. Moreover, in order to ensure an altered IL-6/STAT3 axis as well as FXR deficiency in HCC cells, the gene expression levels of IL-1β, IL-6, STAT3, & FXR were assessed in normal liver cells and compared to that in HepG2 and Huh7 cells. An upregulation of IL-1β, IL-6, & STAT3 besides a downregulation of FXR were observed in HCC cells compared to normal cells.

From a mechanistic perspective, there are some pathways that still need to be deciphered. One of these pathways is the activation of the fibroblast growth factor 4 receptor (FGFR4) by fibroblast growth factor (FGF)15 (in mice) or FGF19 (in humans) following FXR stimulation in the intestine. Despite the beneficial effect of FGF15/19 activation on the liver since they were considered crucial for the regenerative process by protecting against bile acid stress and promoting hepatocyte proliferation^35^, it’s emerging as an important player in hepatic carcinogenesis^36^. Hence, exploring the effect of OCA on FGF15/19 in HCC and investigating whether the antiproliferative effect observed could be related to an altered FGF15/19 level need to be addressed in future studies. Moreover, newly synthesized FXR agonists are yet to be investigated in HCC. For instance, Px-102 and Px-104 are non-steroidal FXR agonists that are still in phase II clinical trials, however, they are more potent than OCA and can protect against bile acid overload and hence have broad spectrum of improvements^37,38^.

Taken together, the results of the present study suggest a potential of the FXR agonist, OCA, for inhibiting HCC proliferation, migration, and invasion *via* interfering with the activation of IL-6/STAT3 signalling pathway. OCA may have decreased STAT3 signalling by abrogating the FXR deficiency and hence the increase in IL-1β & IL-6 as well as increasing the levels of SOCS3, the feedback inhibitor of STAT3. Further studies, however, are required to validate the anticancer effect of OCA in HCC and investigate its potential modulation of the IL-6/STAT3 signalling pathway *in vivo*.
